# Binding mechanism of anti-cancer chemotherapeutic drug mitoxantrone to DNA characterized by magnetic tweezers

**DOI:** 10.1186/s12951-018-0381-y

**Published:** 2018-07-13

**Authors:** Dennis Kreft, Ying Wang, Michael Rattay, Katja Toensing, Dario Anselmetti

**Affiliations:** 1 Experimental Biophysics and Applied Nanoscience, Physics Department, Bielefeld Institute for Nanoscience (BINAS), Bielefeld University, Universitaetsstrasse 25, 33615 Bielefeld, Germany; 2 Baxter Oncology GmbH, Kantstrasse 2, 33790 Halle Westphalia, Germany

**Keywords:** Mitoxantrone, DNA, Magnetic tweezers, Intercalator, Groove binder

## Abstract

**Background:**

Chemotherapeutic agents (anti-cancer drugs) are small cytostatic or cytotoxic molecules that often bind to double-stranded DNA (dsDNA) resulting in modifications of their structural and nanomechanical properties and thus interfering with the cell proliferation process.

**Methods:**

We investigated the anthraquinone compound mitoxantrone that is used for treating certain cancer types like leukemia and lymphoma with magnetic tweezers as a single molecule nanosensor. In order to study the association of mitoxantrone with dsDNA, we conducted force-extension and mechanical overwinding experiments with a sensitivity of 10^−14^ N.

**Results:**

Using this method, we were able to estimate an equilibrium constant of association K_a_ ≈ 1 × 105 M^−1^ as well as a binding site size of n ≈ 2.5 base pairs for mitoxantrone. An unwinding angle of mitoxantrone-intercalation of ϑ ≈ 16° was determined.

**Conclusion:**

Moreover, we observed a complex concentration-dependent bimodal binding behavior, where mitoxantrone associates to dsDNA as an intercalator and groove binder simultaneously at low concentrations and as a mere intercalator at high concentrations.

**Electronic supplementary material:**

The online version of this article (10.1186/s12951-018-0381-y) contains supplementary material, which is available to authorized users.

## Background

Regarding the high morbidity and mortality rate of cancer diseases in the recent decades, the development of cytostatic and cytotoxic chemotherapeutics is highly promoted. Several types of such anti-tumor agents, e.g. anthracycline, bind to DNA polymers in tumor/cancer cells and consequently result in an inhibition of cell growth (cytostatic/antiproliferative activity) or even necrosis (cytotoxic activity). Their heal efficacy depends strongly on the binding mode and the nanomechanism of the DNA-drug interaction. Therefore, a deep and thorough understanding of these biophysical characteristics of chemotherapeutics in the perspective of molecular recognition contributes significantly to the medical regulation and optimization of pharmaceutics.

**Fig. 1:**
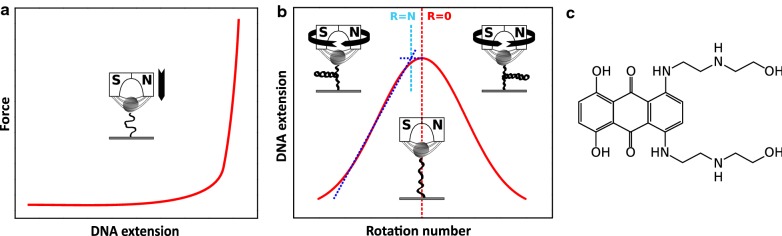
Schematic of the MT assays for **a** extending and **b** overwinding a single dsDNA molecule (hat curve). Blue dashed lines divide the hat curve into two regions where a dsDNA polymer exhibits different torsional behaviors. The rotation number at the transition point is referred to as buckling number (for details see main text); **c** chemical structure of MTX

Here, we focused on an anthraquinone derivative mitoxantrone (MTX, 1,4-dihydroxy-5,8-bis[2-(2-hydroxyethylamino)ethylamino]anthracene-9,10-dione, chemical structure see Fig. 1c [1]). The topoisomerase II-inhibitor MTX was first synthesized in the late 1970s by Zee-Cheng and Cheng and Murdock et al. independently [2–4]. As a promising chemotherapeutics, MTX is broadly used in the treatment of different cancers such as metastatic breast cancer and acute lymphoblastic leukemia as well as of multiple sclerosis [5–9]. Compared to other members of the anthracycline family, MTX has a comparable cytostatic activity but lower cardiotoxicity [10–13]. Besides the medical applications, the binding of MTX to DNA and its corresponding influence on the nanomechanical and structural properties of DNA are still not fully understood. MTX was well-known to bind to DNA as a classical intercalator. However, several publications pointed out an additional groove-binding of MTX [14–20]. The quantifications of the binding mechanism of MTX are also not very consistent. Kapuscinski et al. reported a binding affinity of the MTX-DNA interaction of *K*_*a*_ ~ 10^5^ M^−1^ [21], whereas other research groups estimated the value one order of magnitude higher [15, 18, 22–25]. Furthermore, DNA-untwisting due to MTX-intercalation was hardly studied. In this work, we performed single molecule nanosensor magnetic tweezers (MT) experiments to investigate the association of MTX with dsDNA. By means of extending and overwinding experiments within a force range of 0.005–10 pN, we analyzed the effects of MTX-binding on the nanomechanical and structural properties of dsDNA e.g. elongation, softening and unwinding. As a result, we categorized the MTX-dsDNA association as a complex concentration-dependent bimodal binding.

## Methods

For the performance of MT-experiments (Fig. 1a/b), we used a commercial MT system (PicoTwist, Lyon, France) with a self-made microfluidic flow cell. The experimental setup and flow cell assembly were previously described in detail [26–31]. In brief, the surface of the flow cell was covalently coated with sigmacote (Sigma-Aldrich, Hamburg, Germany) for a homogeneous hydrophobic surface and subsequently functionalized with anti-digoxigenin (200 μg/ml, Roche, Penzberg, Germany). For MT experiments, we prepared λ-dsDNA fragments which were functionalized at one end with several biotins (Biotin-14-dCTP, Metabion, Steinkirchen, Germany) and with several digoxigenins (Dig-11-dUTP, Roche, Penzberg, Germany) at the other end according to a published protocol [29, 32, 33]. The 11.8 kbp fragments, corresponding to a contour length of about 4 µm, were separated by gel electrophoresis. Via the specific bonds, single dsDNA molecules were attached between the anti-dig functionalized surface and streptavidin coated superparamagnetic beads with a diameter of 1 µm (Dynabeads MyOne, Thermo Fisher Scientific, Waltham, USA). As a reference and control for each investigated DNA molecule, we verified its contour- and persistence length by means of stretching experiments and approximation of the force-extension curves to the worm-like-chain (WLC) polymer elasticity model [34, 35]:

1 $$\frac{FP}{{k_{\scriptscriptstyle\text{B}} T}} = \frac{1}{4}\left( {\left( {1 - \frac{d}{L(c)}} \right)^{ - 2} - 1} \right) + \frac{d}{L(c)}$$

Here, *F*, *P*, *L*(*c*), *k*_B_*T* and *d* represent the applied force, dsDNA persistence length, dsDNA contour length as functions of the drug concentration *c*, thermal energy and molecular extension of the dsDNA (end-to-end distance), respectively. Furthermore, we acquired reference “hat curves” via overwinding dsDNA to verify the nick-free structure of probed molecules.

All experiments were performed at 25 °C with MT buffer consisting of 10 mM phosphate buffered saline (PBS, with 137 mM NaCl + 2.7 mM KCl, pH 7.4 @ 25 °C) with 0.1 mg/ml additional bovine serum albumin (BSA, Sigma-Aldrich, Hamburg, Germany) and 0.1% TWEEN 20 (Sigma-Aldrich, Hamburg, Germany) inhibiting possible unspecific bonds. The cytostatics MTX was supplied by Baxter Oncology GmbH (Halle Westphalia, Germany), dissolved in PBS as stock solution (1 mM) and for further experiments diluted with MT buffer to concentrations from 10 nM up to 30 µM. 0.2 nM dsDNA was incubated with MTX for 2 h to reach the thermodynamic equilibrium and subsequently gently flushed into the chamber. MT force-extension experiments were performed with forces up to 10 pN after verifying the thermodynamic equilibrium binding state (data not shown, see Additional file 1). All experiments were repeated with at least 10 individual single molecules for each MTX concentration. Moreover, we replaced the complete flow cell after every statistical measurement series. The data were approximated with the WLC model and the dsDNA contour- and persistence length were fitted. In addition, by applying the transformed noncooperative McGhee-von-Hippel binding model for thermal equilibrium [36–38]:

2 $$\dfrac{\gamma }{c} = K_{a} \dfrac{\Delta x}{{x_{\scriptscriptstyle\text{bp}} }} \cdot \dfrac{{\left( {1 - \dfrac{{n \gamma x_{\scriptscriptstyle\text{bp}} }}{\Delta x}} \right)^{n} }}{{\left( {1 - \dfrac{{\left( {n - 1} \right) \gamma x_{\scriptscriptstyle\text{bp}} }}{\Delta x}} \right)^{n - 1} }}$$

the relation between the fractional elongation of dsDNA *γ* and drug concentration *c* was determined. *K*_*a*_ denotes the equilibrium constant of association for intercalation, Δ*x* is the dsDNA elongation due to one intercalated agent molecule, *x*_bp_ represents the reference distance between two base pairs (*x*_bp_ = 0.34 nm). *n* is the binding site size per drug molecule referring to the average length of base pairs, which are responsible for the intercalation. The fractional elongation *γ* can be expressed as

3 $$\gamma = \frac{{L(c) - L_{0} }}{{L_{0} }}$$

where *L*_0_ is the contour length of a bare dsDNA. The fitting errors of *L*(*c*) and *L*_0_ contribute to the uncertainty of *γ*, Δ*x*, *K*_*a*_ and *n* via propagation of uncertainty. All overwinding experiments were performed with a preset force of 0.2 pN where MTX was immersed with stepwise increasing concentrations.

## Results and discussion

### Extension-experiments

**Fig. 2:**
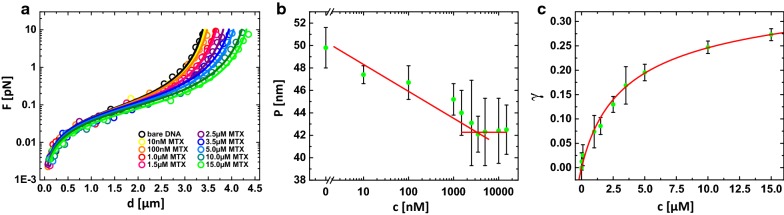
**a** dsDNA extension-experiments with different MTX concentrations. Open circles show the experimental data and solid lines represent the fitting to the WLC model. **b** Persistence length *P* of dsDNA in dependence of drug concentrations (green dots). The zones below and beyond the threshold concentration were approximated by a straight line (< 3 µM) and a zero slope line (> 3 µM), **c** plot of fractional elongation of DNA *γ* with drug concentrations. Green dots show the experimental data that were approximated to the McGhee-von Hippel model (solid line)

We used MT based extension and overwinding experiments to investigate the influence of the MTX association on the nanomechanical properties of dsDNA. Firstly, we conducted stretching experiments while the dsDNA remained in the torsionally relaxed state exposing its maximum end-to-end length. The force-extension curves of MTX-dsDNA mix are presented in Fig. 2a. The contour- and persistence length of the investigated dsDNA molecules were estimated via approximating the data to the WLC-model.

At low MTX concentrations up to 3 µM, we discovered successive shifts of the force-extension curves indicating larger dsDNA contour lengths. Interestingly, at the same time the persistence length decreased from about 50 ± 2 to 42 ± 2 nm. Further increasing the drug concentration, merely an increment of the contour length was detected. At a drug concentration of 15 µM, we found a dsDNA-elongation of 27%. In previous work, we were able to categorize the binding mode of a dsDNA-binding agent by its influence on the nanomechanical properties of the host molecule, i.e. an intercalator elongates the dsDNA virtually without affecting the bending stiffness; in contrast, a groove binder only softens the dsDNA [33]. That leads to the conclusion that MTX-dsDNA association exhibits a concentration-dependent bimodal binding mechanism. Primarily, MTX intercalates and groove-binds to dsDNA simultaneously, i.e. the planar anthraquinone ring interacts with the dsDNA base pairs in both intercalating and groove-like binding modes. Moreover, the aminoethylamino side chains bind electrostatically to the negatively charged phosphate backbones strengthening the MTX-dsDNA interaction. This matches with the results from the earlier reports [14–19, 22, 39–41]. Beyond the threshold concentration of 3 µM, the intercalation becomes dominant. Notably, in the case of bimodal binding, it is still not clear in which groove the electrostatic interaction occurs. Lown et al. and Wang et al. suggested that two aminoethylamino chains fit to the major groove by electrochemical experiments and a high-field 1H-NMR analysis, respectively [14, 18, 20]. In contrast, Mazerski et al. reported a minor-groove association of both side chains [17]. Several other work found that the helically shaped chains of MTX can associate in both grooves. However, the interaction in the minor groove was found less favorable and sequence-selective [15, 16, 19].

### Determination of binding mechanism

In addition, we approximated the fractional elongation data to the non-cooperative McGhee-von Hippel binding model (Fig. 2c) and obtained an elongation per intercalated drug molecule of *∆x* = 0.37 ± 0.02 nm, corresponding to a rise of a B-DNA base pair (0.34 nm). The binding site size *n* was determined as *n* = 2.51 ± 0.11 bp, which is typical for a monointercalator and conforms to the “nearest neighbor exclusion principle” [42–44]. This matches very well with previous results [18, 21, 40] although earlier Kapuscinski et al. also reported a *n*-value of 5 bp for MTX [39]. Analogously, we calculated an equilibrium constant of association of *K*_*a*_ = (0.98 ± 0.06) × 10^5^ M^−1^, which is consistent with the results of Kapuscinski et al. of *K*_*a*_ = 2.5 × 10^5^ M^−1^ [21] but somewhat lower than published by other groups [15, 18, 22–25, 39]. However, since MTX apparently presents a more complex bimodal binding mode, the theoretical model might be of a somewhat limited applicability.

### Overwinding-experiments

**Fig. 3:**
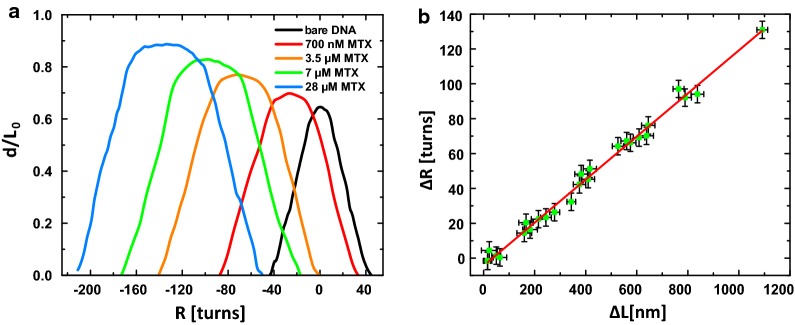
**a** Results of single DNA molecule overwinding experiments with stepwise increased MTX concentration at a preset force of 0.2 pN; **b** plot of the change of the rotation number Δ*R* with corresponding elongation of the DNA contour length Δ*L*. The slope (linear approximation, solid line) allows estimating the unwinding angle per intercalated MTX molecule

In order to determine the unwinding angle of the MTX-intercalation, we performed overwinding-experiments that allowed us to twist individual nick-free dsDNA molecules in a well-defined manner. The pulling force was preset to 0.2 pN. The resulting supercoiling states were recorded as so called “hat curves” (Fig. 3a). At such small forces, a bare dsDNA molecule exhibits a symmetric torsional behavior. The peak positions of these curves describe the rotationally relaxed state of the dsDNA double helix. Starting from here, a hat curve can be divided into two phases (Fig. 1b, blue dashed line). In the first phase, the dsDNA length hardly changes upon twisting where the mechanical torque on dsDNA is released along the double strands. In the second phase, the dsDNA end-to-end distance decreases linearly with the number of added turns where plectonemes are formed [33, 45–47]. The buckling number *N* defines the crossover regime of these two phases (Fig. 1b). In contrast, a multiple rotation of a nicked dsDNA molecule causes no under- or overwinding since the single strand can rotate around the phosphodiester bond in idle state [48]. Such structural characteristics of dsDNA polymers can be used to study dsDNA unwinding induced by drug-intercalation. The local unwinding generates positive supercoilings which can be detected as a sudden dsDNA length decrement or a shift of hat curves [31, 33, 46, 49–52].

The overwinding experiments were recorded with added MTX concentrations up to 28 µM. The hat curve of bare dsDNA was taken as reference (black curve, Fig. 3a). By increasing the MTX concentration, an obvious shift of the hat curves to negative rotation numbers was observed, indicating a DNA unwinding and further supporting the intercalative binding mode of MTX [44]. In addition, a height increment of the hat curves implies an intercalation induced dsDNA elongation that is fully consistent with our extension experiments [44].

Moreover, we evaluated and plotted the change in the rotation number Δ*R* and the elongation of the dsDNA contour length Δ*L* (Fig. 3b). The linear approximation of the data gave us a slope of 0.121 ± 0.002 turns/nm.

According to the following correlation

4 $$\theta = \frac{\Delta R}{\text{number of bound MTX}} = \frac{\Delta R}{\Delta L} \cdot \Delta x$$

the unwinding angle per intercalated MTX molecule *θ* can be calculated combining the slope of the linear fit and the previously determined elongation per drug molecule Δ*x* [31, 33, 49]. As a result, we obtained an unwinding angle of 0.045 ± 0.003 turns/drug corresponding to* θ* = 16 ± 1°/drug. This result is in full accordance with the reported value of Lown et al. from their independent viscosity and topoisomerase assays (17.5°, [15]), but considerably lower compared to the report from Kapuscinski et al. (26.5°, [39]).

## Conclusion

In summary, we investigated the nanomechanical binding mechanism of MTX to dsDNA at room temperature in PBS buffer by employing a MT single molecule nanosensor. As a conventional mono-intercalator, MTX displayed a fast equilibrium assembly compared with bis-intercalators and threading intercalators [53–58]. By means of extending and overwinding individual DNA molecules, we observed an elongation, softening and untwisting of the DNA double helix upon MTX binding in a concentration dependent manner. Based on earlier findings [33], we identified a bimodal association mode, i.e. MTX exhibits simultaneously an intercalative and groove-binding behavior. In addition, we determined a threshold concentration of 3 µM at which the primary bimodal association declines and mere intercalation becomes dominant. Furthermore, we estimated a binding site size of *n* ≈ 2.5 bp, which corresponds to the results of previous reports (*n* = 2.6–3.0 bp) [18, 21, 40]. An elongation of Δ*x* ≈ 0.37 nm induced by each drug molecule was estimated, which is typical for a mono-intercalator, since the bond between the drug molecule and DNA base pairs is stabilized through π-stacking. Moreover, we found that each intercalated MTX molecule unwinds the native DNA helix with an angle *θ* of about 16°, compensating the elongation-induced tension. Finally, the equilibrium constant of association of MTX-dsDNA interaction was determined to be about *K*_*a*_ ≈ 1 × 10^5^ M^−1^, which is significantly lower than in previous reports [15, 18, 22–25, 39]. However, other anthraquinone derivates like DRAQ5 were found to occupy a similar binding affinity to DNA [33, 59–63]. The results of this work help to further characterize and quantify the biophysical binding mode of mitoxantrone to dsDNA and in turn support the medical regulation processes.

## Additional file

**Additional file 1.** Force clamp experiments of dsDNA molecule with 3 µM MTX at different forces. dsDNA was incubated with 3 µM Mitoxantrone for 2 h in the relaxed state. The forces 0.1, 1, 5 and 10 pN were successively applied to the bead so that the DNA molecule was stretched. After a delay of 10 s, which was as well included in the force-extension measurements, the DNA extensions were recorded as a function of time. The constant DNA lengths in a large time scale (10 min) indicate that the mitoxantrone already equilibrated its association to the DNA before the force measurements were taken and displayed a fast equilibrium assembly. Here, *d*/*L*_0_ is the normalized end-to-end distance of the DNA molecule and *L*_0_ represents the DNA contour length in the absence of mitoxantrone.

## References

[1] National Center for Biotechnology Information. https://pubchem.ncbi.nlm.nih.gov/compound/4212. PubChem Compound Database. Accessed 2018 Jan 29.

[2] Zee-Cheng RKY, Cheng CC (1978). Antineoplastic agents. Structure–activity relationship study of bis(substituted aminoalkylamino)anthraquinones. J Med Chem.

[3] Murdock KC, Child RG, Fabio PF, Angier RB, Wallace RE, Durr FE (1979). Antitumor Agents. 1. 1,4-Bis[(aminoalkyl)amino]-9,10-anthracenediones. J Med Chem.

[4] Wu C-C, Li Y-C, Wang Y-R, Li T-K, Chan N-L (2013). On the structural basis and design guidelines for type II topoisomerase-targeting anticancer drugs. Nucleic Acids Res.

[5] Holmes FA, Yap H-Y, Esparza L, Buzdar AU, Hortobagyi GN, Blumenschein GR (1984). Mitoxantrone, cyclophosphamide, and 5-fluorouracil in the treatment of hormonally unresponsive metastatic breast cancer. Semin Oncol.

[6] Smith PJ, Morgan SA, Fox ME, Watson JV (1990). Mitoxantrone-DNA binding and the induction of topoisomerase II associated DNA damage in multi-drug resistant small cell lung cancer cells. Biochem Pharmacol.

[7] Thomas X, Archimbaud E (1997). Mitoxantrone in the treatment of acute myelogenous leukemia: a review. Hematol Cell Ther.

[8] Scott LJ, Figgitt DP (2004). Mitoxantrone: a review of its use in multiple sclerosis. CNS Drugs.

[9] Parker C, Waters R, Leighton C, Hancock J, Sutton R, Moorman AV (2010). Effect of mitoxantrone on outcome of children with first relapse of acute lymphoblastic leukaemia (ALL R3): an open-label randomised trial. Lancet.

[10] Posner LE, Dukart G, Goldberg J, Bernstein T, Cartwright K (1985). Mitoxantrone: an overview of safety and toxicity. Invest New Drugs.

[11] White RJ, Durr FE (1985). Development of mitoxantrone. Invest New Drugs.

[12] Alderton PM, Green MD (1992). Comparative study of doxorubicin, mitoxantrone, and epirubicin in combination with ICRF-187 (ADR-529) in a chronic cardiotoxicity animal model. Cancer Res.

[13] Koutinos G, Stathopoulos GP, Dontas I, Perrea-Kotsarelis D, Couris E, Karayannacos PE (2002). The effect of doxorubicin and its analogue mitoxantrone on cardiac muscle and on serum lipids: an experimental study. Anticancer Res.

[14] Lown JW, Hanstock CC (1985). High Field 1H-NMR analysis of the 1:1 intercalation complex of the antitumor agent mitoxantrone and the DNA duplex [d(CpGpCpG)]2. J Biomol Struct Dyn.

[15] Lown JW, Morgan AR, Yen SF, Wang YH, Wilson WD (1985). Characteristics of the binding of the anticancer agents mitoxantrone and ametantrone and related structures to deoxyribonucleic acids. Biochemistry.

[16] Chen K-X (1986). A theoretical investigation on the sequence selective binding of mitoxantrone to double-stranded tetranucleotides. Nucleic Acids Res.

[17] Mazerski J, Martelli S, Borowski E (1998). The geometry of intercalation complex of antitumor mitoxantrone and ametantrone with DNA: molecular dynamics simulations. Acta Biochim Pol.

[18] Wang S, Peng T, Yang CF (2003). Electrochemical determination of interaction parameters for DNA and mitoxantrone in an irreversible redox process. Biophys Chem.

[19] Parker BS, Buley T, Evison BJ, Cutts SM, Neumann GM, Iskander MN (2004). A molecular understanding of mitoxantrone-DNA adduct formation. J Biol Chem.

[20] Varadwaj P, Misra K, Sharma A, Kumar R (2010). Mitoxantrone: an agent with promises for anticancer therapies. Electron J Biol.

[21] Kapuscinski J, Darzynkiewicz Z (1985). Interactions of antitumor agents Ametantrone and Mitoxantrone (Novatrone) with double-stranded DNA. Biochem Pharmacol.

[22] Rosenberg LS, Carvlin MJ, Krugh TR (1986). The antitumor agent mitoxantrone binds cooperatively to DNA: evidence for heterogeneity in DNA conformation. Biochemistry.

[23] Zagotto G, Sissi C, Palumbo G (2004). Aminoacyl-analogues of mitoxantrone as novel DNA-damaging cytotoxic agents. Arkivoc..

[24] Hajihassan Z, Rabbani-Chadegani A (2009). Studies on the binding affinity of anticancer drug mitoxantrone to chromatin, DNA and histone proteins. J Biomed Sci.

[25] Bhattacharyya J, Basu A, Suresh Kumar G (2014). Intercalative interaction of the anticancer drug mitoxantrone with double stranded DNA: a calorimetric characterization of the energetics. J Chem Thermodyn.

[26] Strick TR, Allemand JF, Bensimon D, Bensimon A, Croquette V (1996). The elasticity of a single supercoiled DNA molecule. Science.

[27] Strick TR, Allemand JF, Bensimon D, Croquette V (1998). Behavior of supercoiled DNA. Biophys J.

[28] Vilfan ID, Lipfert J, Koster DA, Lemay SG, Dekker NH, van Hinterdorfer O (2009). Magnetic tweezers for single-molecule experiments. Handbook single-molecule biophys.

[29] Jany T, Moreth A, Gruschka C, Sischka A, Spiering A, Dieding M (2015). Rational design of a cytotoxic dinuclear Cu_2_ complex that binds by molecular recognition at two neighboring phosphates of the Dna backbone. Inorg Chem.

[30] Glaser T, von Mollard GF, Anselmetti D (2016). Rational design of dinuclear complexes binding at two neighboring phosphate esters of DNA. Inorganica Chim Acta.

[31] Wang Y, Schellenberg H, Walhorn V, Toensing K, Anselmetti D (2017). Binding mechanism of PicoGreen to DNA characterized by magnetic tweezers and fluorescence spectroscopy. Eur Biophys J.

[32] Cheng W. Protocol to generate half Lambda DNA for optical/magnetic tweezer. 2006. p. 6–9. http://tweezerslab.unipr.it/cgi-bin/mt/documents.pl/Show?_id=ab03&sort=DEFAULT&search=&hits=23. Accessed 2008 Sept 5.

[33] Wang Y, Sischka A, Walhorn V, Tönsing K, Anselmetti D (2016). Nanomechanics of fluorescent DNA Dyes on DNA investigated by magnetic tweezers. Biophys J.

[34] Bustamante C, Marko JF, Siggia ED, Smith S (1994). Entropic elasticity of X-phage DNA. Science.

[35] Bouchiat C, Wang MD, Allemand JF, Strick T, Block SM, Croquette V (1999). Estimating the persistence length of a worm-like chain molecule from force-extension measurements. Biophys J.

[36] McGhee JD, von Hippel PH (1974). Theoretical aspects of DNA-protein interactions: co-operative and non-co-operative binding of large ligands to a one-dimensional homogeneous lattice. J Mol Biol.

[37] Vladescu ID, McCauley MJ, Nuñez ME, Rouzina I, Williams MC (2007). Quantifying force-dependent and zero-force DNA intercalation by single-molecule stretching. Nat Methods.

[38] Kleimann C, Sischka A, Spiering A, Tönsing K, Sewald N, Diederichsen U (2009). Binding kinetics of bisintercalator triostin a with optical tweezers force mechanics. Biophys J.

[39] Kapuscinski J, Darzynkiewicz Z (1981). Interactions of a new antitumor agent, 1,4-dihydroxy-5, 8-bis-ethyl] amino]-9, 10-anthracenedione, with nucleic acids. Biochem Pharmacol.

[40] Foye WO, Vajragupta O, Sengupta SK (1982). DNA-binding specificity and RNA polymerase inhibitory activity of Bis(aminoalky1)anthraquinones and Bis(methy1thio)vinylquinolinium Iodides. J Pharm Sci.

[41] Tang L (2006). Study on the interaction of anticancer drug mitoxantrone with DNA by fluorescence and Raman spectroscopies. Chinese Opt Lett.

[42] Crothers DM (1968). Calculation of binding isotherms for heterogeneous polymers. Biopolymers.

[43] Müller W, Crothers DM (1968). Studies of the binding of actinomycin and related compounds to DNA. J Mol Biol.

[44] Williams LD, Eglifgb M, Gao Q, Ricfgh A, Sarma RH, Sarma MH (1992). DNA intercalation: helix unwinding and neighbor-exclusion. Structure functional nucleic acids.

[45] Strick TR, Dessinges M-N, Charvin G, Dekker NH, Allemand J-F, Bensimon D (2003). Stretching of macromolecules and proteins. Reports Prog Phys.

[46] Salerno D, Brogioli D, Cassina V, Turchi D, Beretta GL, Seruggia D (2010). Magnetic tweezers measurements of the nanomechanical properties of DNA in the presence of drugs. Nucleic Acids Res.

[47] Rutkauskas M, Krivoy A, Szczelkun MD, Rouillon C, Seidel R (2017). Single-molecule insight into target recognition by CRISPR–Cas complexes.

[48] Wang Y, van Merwyk L, Toensing K, Walhorn V, Anselmetti D, Fernàndez-Busquets X (2017). Biophysical characterization of the association of histones with single-stranded DNA. Biochim Biophys Acta Gen Subj.

[49] Guenther K, Mertig M, Seidel R (2010). Mechanical and structural properties of YOYO-1 complexed DNA. Nucleic Acids Res.

[50] Lipfert J, Klijnhout S, Dekker NH (2010). Torsional sensing of small-molecule binding using magnetic tweezers. Nucleic Acids Res.

[51] Szczelkun MD, Tikhomirova MS, Sinkunas T, Gasiunas G, Karvelis T, Pschera P (2014). Direct observation of R-loop formation by single RNA-guided Cas9 and Cascade effector complexes. Proc Natl Acad Sci.

[52] Rutkauskas M, Sinkunas T, Songailiene I, Tikhomirova MS, Siksnys V, Seidel R (2015). Directional R-loop formation by the CRISPR–cas surveillance complex cascade provides efficient off-target site rejection. Cell Rep.

[53] Krishnamoorthy CR, Yen SF, Smith JC, Lown JW, Wilson WD (1986). Stopped-flow kinetic analysis of the interaction of anthraquinone anticancer drugs with calf thymus DNA, poly[d(G-C)].cntdot.poly[d(G-C)], and poly[d(A-T)].cntdot.poly[d(A-T)]. Biochemistry.

[54] Hayashi M, Harada Y (2007). Direct observation of the reversible unwinding of a single DNA molecule caused by the intercalation of ethidium bromide. Nucleic Acids Res..

[55] Murade CU, Subramaniam V, Otto C, Bennink ML (2009). Interaction of oxazole yellow dyes with DNA studied with hybrid optical tweezers and fluorescence microscopy. Biophys J Biophys Soc.

[56] Almaqwashi AA, Paramanathan T, Lincoln P, Rouzina I, Westerlund F, Williams MC (2014). Strong DNA deformation required for extremely slow DNA threading intercalation by a binuclear ruthenium complex. Nucleic Acids Res.

[57] Biebricher AS, Heller I, Roijmans RFH, Hoekstra TP, Peterman EJG, Wuite GJL (2015). The impact of DNA intercalators on DNA and DNA-processing enzymes elucidated through force-dependent binding kinetics. Nat Commun.

[58] Almaqwashi AA, Paramanathan T, Rouzina I, Williams MC (2016). Mechanisms of small molecule—DNA interactions probed by single-molecule force spectroscopy. Nucleic Acids Res.

[59] Costantino L, Guarino G, Ortona O, Vitagllano V (1984). Acridine orange association equilibrium in aqueous solution. J Chem Eng Data.

[60] Roche CJ, Berkowitz D, Sulikowski GA, Danishefsky SJ, Crothers DM (1994). Binding affinity and site selectivity of daunomycin analogues. Biochemistry.

[61] Breslin DT, Yu C, Ly D, Schuster GB (1997). Structural modification changes the DNA binding mode of cation- substituted anthraquinone photonucleases: association by intercalation or minor groove binding determines the DNA cleavage efficiency. Biochemistry.

[62] McKnight RE, Zhang J, Dixon DW (2004). Binding of a homologous series of anthraquinones to DNA. Bioorganic Med. Chem. Lett..

[63] Wang Y, Schellenberg H, Walhorn V, Toensing K, Anselmetti D (2017). Binding mechanism of fluorescent dyes to DNA characterized by magnetic tweezers. Mater Today Proc.

