# Correction to: Binding mechanism of anti-cancer chemotherapeutic drug mitoxantrone to DNA characterized by magnetic tweezers

**DOI:** 10.1186/s12951-018-0430-6

**Published:** 2019-02-08

**Authors:** Dennis Kreft, Ying Wang, Michael Rattay, Katja Toensing, Dario Anselmetti

**Affiliations:** 1 Experimental Biophysics and Applied Nanoscience, Physics Department, Bielefeld Institute for Nanoscience (BINAS), Bielefeld University, Universitaetsstrasse 25, 33615 Bielefeld, Germany; 2 Baxter Oncology GmbH, Kantstrasse 2, 33790 Halle Westphalia, Germany

## Correction to: J Nanobiotechnol (2018) 16:56 10.1186/s12951-018-0381-y

Following publication of this article [1] we found a typographical error in the results reported in the abstract. The corrected sentences should read as below:

Using this method, we were able to estimate an equilibrium constant of association *K*_*a*_ ≈ 1 × 10^5^ M^−1^ as well as a binding site size of *n* ≈ 2.5 base pairs for mitoxantrone. An unwinding angle of mitoxantrone-intercalation of ***ϑ*** ≈ 16° was determined. The original article has been updated.
