# Supplementary figures and images for: Binding mechanism of anti-cancer chemotherapeutic drug mitoxantrone to DNA characterized by magnetic tweezers
Source: J Nanobiotechnology. 2018 Jul 13;16:56. doi: 10.1186/s12951-018-0381-y (PMC6043947; doi:10.1186/s12951-018-0381-y)

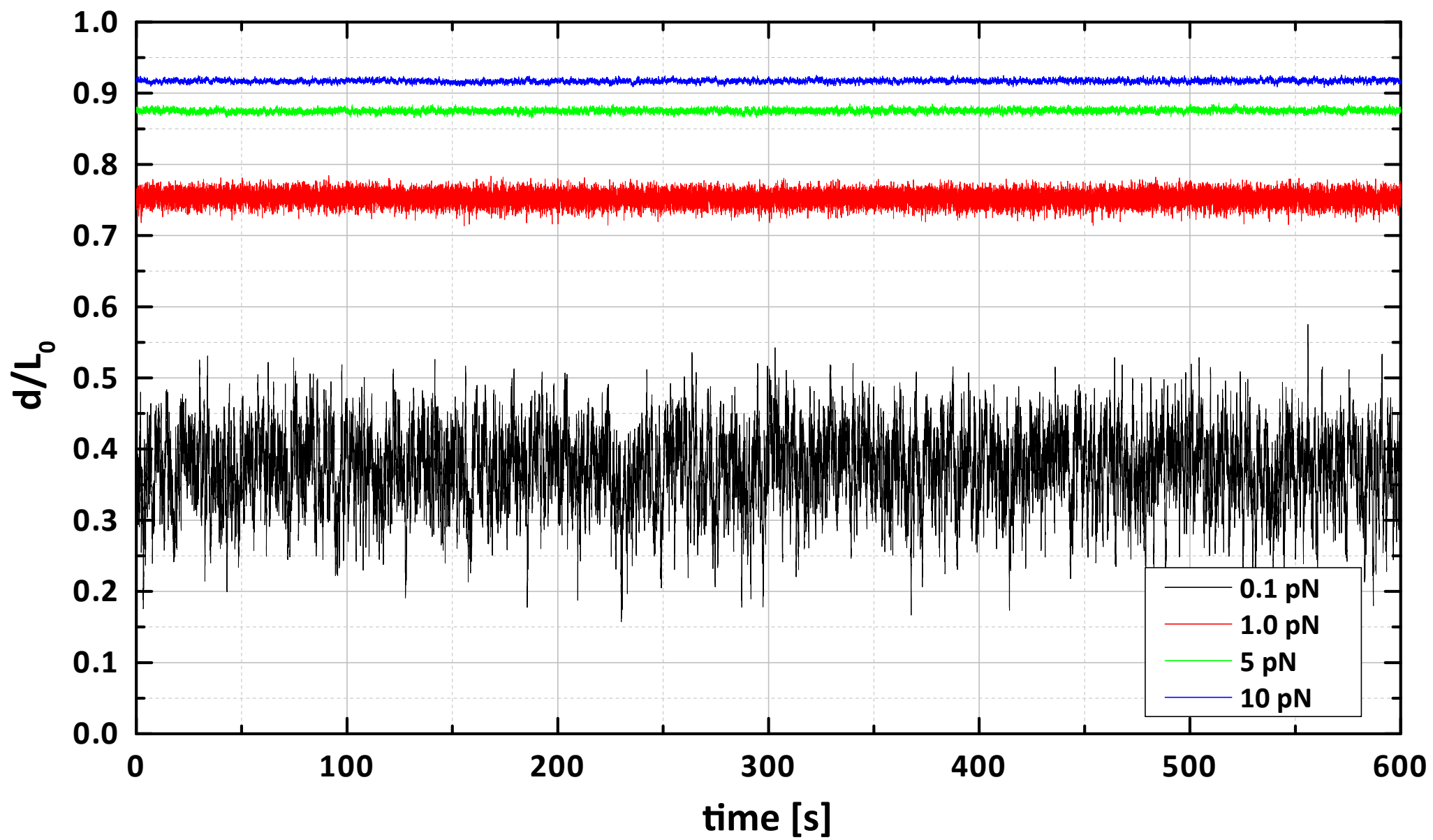

Supplement: Supplementary file 1 — Additional file 1. Force clamp experiments of dsDNA molecule with 3 µM MTX at different forces. dsDNA was incubated with 3 µM Mitoxantrone for 2 h in the relaxed state. The forces 0.1, 1, 5 and 10 pN were successively applied to the bead so that the DNA molecule was stretched. After a delay of 10 s, which was as well included in the force-extension measurements, the DNA extensions were recorded as a function of time. The constant DNA lengths in a large time scale (10 min) indicate that the mitoxantrone already equilibrated its association to the DNA before the force measurements were taken and displayed a fast equilibrium assembly. Here, d/L0 is the normalized end-to-end distance of the DNA molecule and L0 represents the DNA contour length in the absence of mitoxantrone. [file 12951_2018_381_MOESM1_ESM.pdf]
